# Synthetic biology in the view of European public funding organisations

**DOI:** 10.1177/0963662510393624

**Published:** 2012-02

**Authors:** Lei Pei, Sibylle Gaisser, Markus Schmidt

**Keywords:** ELSI, public funding, science funding, science policy, synthetic biology

## Abstract

We analysed the decisions of major European public funding organisations to fund or not to fund synthetic biology (SB) and related ethical, legal and social implication (ELSI) studies. We investigated the reaction of public organisations in six countries (Austria, France, Germany, the Netherlands, Switzerland and the UK) towards SB that may influence SB’s further development in Europe. We examined R&D and ELSI communities and their particular funding situation. Our results show that the funding situation for SB varies considerably among the analysed countries, with the UK as the only country with an established funding scheme for R&D and ELSI that successfully integrates these research communities. Elsewhere, we determined a general lack of funding (France), difficulties in funding ELSI work (Switzerland), lack of an R&D community (Austria), too small ELSI communities (France, Switzerland, Netherlands), or difficulties in linking existing communities with available funding sources (Germany), partly due to an unclear SB definition.

## 1. Introduction

In 2005 the European Commission (EC) convened a high-level expert group to discuss and define a new science and technology field termed synthetic biology (SB). The group agreed that “Synthetic biology is the engineering of biology: the synthesis of complex, biologically based (or inspired) systems which display functions that do not exist in nature” and concluded that “This engineering perspective may be applied at all levels of the hierarchy of biological structures – from individual molecules to whole cells, tissues and organisms. In essence, synthetic biology will enable the design of ‘biological systems’ in a rational and systematic way” (EC, 2005). This forward-looking and promising expert statement was in line with the EC pathfinder initiative in the 6th framework programme (FP6) called NEST: New and Emerging Technologies.^1^ NEST came up with calls for synthetic biology-related proposals (both science and supporting activities) and ultimately funded 18 synthetic biology projects in Europe, with a total budget of over €32 million (Table 1) (EC, 2007). Although the EC is funding four new synthetic biology-related projects in FP7 (two in science and two in humanities) it has so far refrained from providing strong support such as in FP6-NEST. The main reason for that was that with the end of FP6 and the onset of FP7, the EC decided to discontinue the NEST funding programme. According to information from the EC, however, the NEST programme was meant to stimulate a research community in Europe, with the aim that national funding agencies should later continue to fund scientific projects in that area.

**Table 1:** All synthetic biology projects and their funding that were supported by the European Commission’s 6th framework programme NEST

Project acronym	Total project cost (in €1000)	EC contribution (in €1000)
BIOMODULAR H2	2482	1998
BIONANO-SWITCH	2680	1992
CELLCOMPUT	1716	1716
COBIOS*	2582	2064
EMERGENCE*	1520	1520
EUROBIOSYN	2742	1260
FuSyMEM	1400	1400
HIBLIB	3585	1999
NANOMOT	2400	2250
NEONUCLEI	2464	1949
NETSENSOR	1989	1320
ORTHOSOME	1587	982
PROBACTYS	2541	1900
SYNBIOCOM*	264	264
SYNBIOLOY*	226	226
SYNBIOSAFE*	245	236
SYNTHCELLS	1804	1420
TESSY*	232	232
**Total**	**32459**	**24728**

* Projects that are partly or fully dedicated to societal aspects, technology assessment, education or community building.

*Source*: EC, 2007.

### Aim of the study

Our study was designed to determine whether the European national funding agencies have, since the end of the NEST programme, actually taken up synthetic biology in their portfolio as was envisaged by the EC. The aim was to identify the funding situation and the development strategies for synthetic biology in Europe based on several representative European countries. We wanted to know how European funding agencies define and understand synthetic biology, whether they provide specific resources for its research, whether interdisciplinary projects can be funded and whether ethical, legal and societal issues (ELSI) are taken into account when providing funding support. Our results shed light on whether and how synthetic biology has been received and interpreted by major European funding agencies and how their support could shape the future of synthetic biology in Europe.

## 2. Methodology

**Figure 1.**
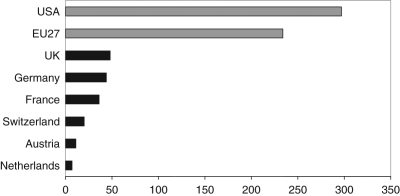
Number of synthetic biology-related PubMed publications (up to November 2009). Search results from GoPubMed using the search terms: “synthetic biology” OR “biological circuit” OR “artificial cell” OR “minimal genome” OR “artificial system” OR “artificial ecosystem” OR “XNA” (xeno nucleic acids). With these keywords, the US is the world’s leader in publications on synthetic biology, followed by Europe (EU27). The six countries selected in our study altogether account for 70.9% of all EU27 publications in synthetic biology. *Source*: GoPubMed, data until 3 November 2009.

**Figure 2.**
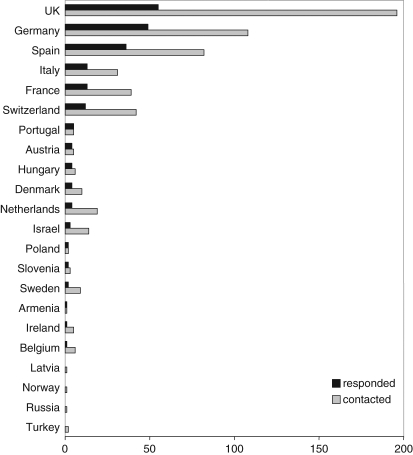
Number of identified scientists working on synthetic biology. A total of 588 European scientists working in synthetic biology-related areas were contacted to participate in a survey on synthetic biology, carried out by the TESSY project. Of the 211 who responded, 130 (or 61.6%) were from the UK, Germany, France, Switzerland, Austria or the Netherlands. *Source*: Gaisser et al 2009

A survey study with interviews with representatives of different public funding organisations from six European countries was conducted from September to October 2009. The structure of the questionnaire was based on a previous study on bioethics in synthetic biology (Ganguli-Mitra et al., 2009). Our intention was to obtain information from enough European countries to cover over 60% of the synthetic biology-related research and ELSI research in Europe – in relation to the number of publications (using GoPubMed^2^) and number of (self-selected) scientists (Figures 1 and 2).

Another requirement was that the selected countries also be represented in the pan-European ESF (European Science Foundation) known as EUROSYNBIO, which was supported by 11 European countries plus Turkey.^3^ We finally chose Germany, the United Kingdom, France (which did not support the ESF call), Switzerland for their important role in European synthetic biology research, and the Netherlands and Austria for their contribution to synthetic biology-related ELSI research. We scheduled 11 interviews with representatives of the national funding agencies of the six selected countries (by phone, approximately 30 minutes per interview). The interviews were conducted with the persons in charge of biotechnology and/or genome science funding at the:

- German Research Foundation (Deutsche Forschungsgemeinschaft, DFG)
- Federal Ministry for Education and Research (Bundesministerium für Bildung und Forschung, BMBF)
- Biotechnological and Biological Sciences Research Council (BBSRC)
- Engineering and Physical Sciences Research Council (EPSRC)
- National Center for Scientific Research (Centre National de la Recherche Scientifique, CNRS)
- French National Research Agency (L’Agence nationale de la recherché, ANR)
- SystemsX.ch (launched as the Swiss Initiative in Systems Biology)
- Austrian Research Promotion Agency’s (FFG) GEN-AU ELSI programme
- Austrian Science Fund (FWF)
- Netherlands Organisation for Scientific Research (NWO)
- Dutch Technology Foundation STW.

Although our interview partners play a key role in the funding agencies, it was made clear that their view does not necessarily represent the official point of view of their organisation. We promised anonymity to the interview partners for later publications, thus we did not add names or positions to the selected quotations in this article.

In addition we searched for online background information about the activities of the funding agencies. Nonetheless, our results are predominantly based on the interviews.

## 3. Results

The results of our work are presented in the following paragraphs, country by country, in order to demonstrate the bandwidth in funding activities. The differences in definitions of synthetic biology are compiled in Table 2. All interviewees associated synthetic biology with biocircuits and metabolic engineering, as well as with an enlarged genetic alphabet and DNA with a chemically different backbone. For other subfields such as “creation of life” and “minimal genome,” less agreement was found.

**Table 2.** Definitions of synthetic biology by interviewed funding representatives

	AT-1	AT-2	CH-1	DE-1	DE-2	FR-1	FR-2	NL-1	NL-2	UK-1	UK-2
**Biocircuits using standard biological parts**	+	+	+	+	+	+	+	+	?	+	+
**Biocircuits without standard biological parts?**	+	+	+	+	+	+	+	+	?	+	+
Engineering cells to produce fine chemicals	+	−	+	+	+	+	+	+	+	+	+
Creating artificial life	−	+	+	−	−	+	+	−	?	+	+
Computer software for biocircuit design	−	+	+	+	+	+	+	+	−	+	−
Artificial ecosystems	−	−	+	?	?	+	+	−	−	+	−
**Enlarged genetic alphabet**	+	+	+	+	+	+	+	+	+	+	+
**DNA with chemically different backbone**	+	+	+	+	+	+	+	+	+	+	+
Minimal genome	−	−	+	+	+	+	+	+	?	+	+
Understanding the origin of life	+	−	−	+	+	+	+	−	+	+	+

Several subfields of synthetic biology were presented to interviewees: “+” means that it was accepted as part of synthetic biology, “–” means that it was not, “?” means don’t know. AT-1: first interviewee Austria, AT-2: second interviewee Austria, CH: Switzerland, DE: Germany, FR: France; NL: Netherlands; UK: United Kingdom. Areas of high agreement are shown in bold.

### National funding organizations

#### Germany

In Germany, two major national funding organisations account for most of the public funding in emerging fields of life sciences. The German Research Foundation (Deutsche Forschungsgemeinschaft, DFG) promotes research at universities and other publicly financed research institutions in Germany in all branches of science and the humanities by a bottom-up approach (responsive-mode). In turn, the Federal Ministry for Education and Research (Bundesministerium für Bildung und Forschung, BMBF) adopts a more applied research funding strategy. Both the DFG and the BMBF are already actively involved in considerations fostering synthetic biology. Other German funding organisations such as the Volkswagen Foundation, the German National Environment Foundation (Deutsche Bundesstiftung Umwelt, DBU) and the Bertelsmann Foundation have not yet entered the field of synthetic biology.

The DFG co-organised an expert consultation, implying the need for a clear discussion and clarification of ethical issues associated with the subtopic “creating artificial life.” Therefore, the DFG and BMBF support scientific research projects on the ethical, legal and economic aspects of health research and bioscientific research. ^4^

Up to January 2010, the DFG has not funded any project with synthetic biology in the title. A number of projects, however, have a potential linkage to synthetic biology. Additionally, the DFG funds the cluster of excellence “Centre for Biological Signalling Studies” (bioss) at the University of Freiburg with €32.5 million for 5 years (2007–2011/12). Approximately one fifth of the bioss activities in Freiburg are linked to synthetic biology. Additionally, the DFG funds one Emmy-Noether-Scholar in the field of SB (expansion of the genetic code). As outlined above, the DFG funds research via a bottom-up approach, i.e. “any research idea can be submitted and will be evaluated according to its scientific quality. Thus, the DFG has an efficient means to fund new ideas such as synthetic biology if they are of excellent scientific quality” as summarised by a DFG expert who was involved in the development of the DFG strategy in the field of synthetic biology.

So far the BMBF has not funded projects with a direct link to synthetic biology, but there is a strong interest in this field on the part of the BMBF. In the past the BMBF has taken the risk involved in funding emerging sciences and technologies, such as systems biology. This field would probably enjoy similar support if the scientific community were to agree on the direction of synthetic biology.

On the level of federal states, the German state of Hessen recently announced a €21 million funding for the synthetic microbiology research cluster “SYNMIKRO” between the University of Marburg and the Max Planck Institute for terrestrial microbiology. SYNMIKRO receives the highest research funding ever granted by Hessen.^5^

In the view of German funders, the synthetic biology community is still small. One interviewee argued “for the size of the synthetic biology community and the number of projects in this field, the money currently available is appropriate, though the sum is small.” However, the expert observed a continuous growth of the synthetic biology community during the last 3 years. “If this growth continues there may be the risk of funding shortfall in the future,” noted the interviewee.

As outlined by the DFG, there is no restriction in specific topics for research funding. Accordingly, funding depends upon scientific quality rather than specific predefined topics, and a plethora of different topics could be submitted and will be funded if they convince the reviewers. The BMBF sees a clear need for accompanying measures in the field of ELSI and outlined that the policy task – as envisaged under the BMBF biotechnology programme – is to combine innovations with social responsibility.^6^

#### United Kingdom

In the United Kingdom, national research funding is organised in seven Research Councils that work together as Research Councils UK (RCUK). Funding activities in synthetic biology are undertaken mainly through the Biotechnological and Biological Sciences Research Council (BBSRC) and the Engineering and Physical Sciences Research Council (EPSRC). “BBSRC and EPSRC support synthetic biology through individual responsive mode grants and core-related research in areas such as systems biology, bioengineering and bionanotechnology” as outlined by one interviewee.

Additionally, four UK Research Councils (BBSRC, EPSRC, ESRC and AHRC) have come together to fund seven research community networks in synthetic biology led by UK universities with a total of nearly €1 million (= £0.9 million). BBSRC currently spends around €20.8 million (= £19 million)^7^ a year on projects in synthetic biology and core-related research areas. EPSRC spent approximately €3.2 million for sign-post activities, e.g. in synthetic biology.^8^ In spring 2009, EPSRC and the US National Science Foundation (NSF) invited submissions on expressions of interest on a joint call on “New Directions in Synthetic Biology Details” and subsequently allocated about €6 million (= £5.5 million) for five research projects.^9^ Finally, EPSRC is funding the Centre for Synthetic Biology and Innovation at Imperial College, London, with €5.3 million (= £4.8 million) between 2009 and 2014. The Medical Research Council (MRC) provided over €1.8 million (= £1.6 million) for research on synthetic biology in 2007/08. The interviewee of MRC mentioned that “they would also consider funding synthetic biology via response-mode [bottom-up approach] but so far MRC did not receive many applications in this scientific area.”

Against the background of a broad awareness of synthetic biology in the UK Research Councils, as well as joint activities and the involvement of other actors including the Royal Society^10^ and the Wellcome Trust,^11^ it can be concluded that the funding situation in the UK is good. Synthetic biology is among the topics that should be fostered according to BBSRC’s strategic plan 2010–2015.^12^ By means of different types of measure, this approach addresses the full range of levels from research funding, community building, networking and education to public dialogue. A report on a public dialogue process regarding synthetic biology was recently published by BBSRC and EPSRC (Bhattachary et al., 2010). The dialogue was intended to help frame the issues and promote a broader debate on SB. Still, BBSRC and EPSRC emphasise the need for the analysis of ELSI in parallel to scientific research and development in synthetic biology. For example, EPSRC encouraged, in its latest call on synthetic biology, investigation of ethics and societal issues concurrently with scientific research. Additionally, BBSRC’s Bioscience for Society Strategy Panel has considered the ethical, legal and societal issues raised by synthetic biology by establishing a Working Group to identify key factors relevant to effecting constructive public engagement. BBSRC has also commissioned an independent review of the UK’s position in synthetic biology and of the key societal issues raised by new research capabilities.^13^

#### France

The French National Research Agency (L’Agence nationale de la recherché, ANR) and the National Centre for Scientific Research (Centre National de la Recherche Scientifique, CNRS) are the two most important organisations active in promoting the life sciences in France. Theoretically, synthetic biology could be funded with the Call for Proposal (CFP) for green and white chemistry, complex systems, and within the non-thematic calls. No dedicated CFP for synthetic biology has been issued by ANR. Research proposals on synthetic biology could be submitted through CFP from the Blanc programme for all research fields, the National Bioenergies Research Programme for second- and third-generation biofuels, and the Emerging Scientific Challenges and “Memory” Key Project for new needs from emerging fields, which includes synthetic biology. So far, one publication on synthetic biology has been produced that was supported by the Blanc programme (Mingardon et al., 2007) and several ongoing projects are also supported by this programme.^14^ As with the situation of ANR, no dedicated scheme for synthetic biology has been defined by CNRS. Synthetic biology-related projects are funded by CNRS through an interdisciplinary programme of research. Compared to its annual budget of €3.36 billion for fiscal year 2009,^15^ the budget for a project funded by the interdisciplinary programme of CNRS is extremely small: only €50,000 over a period of 1 year. To date, only two synthetic biology projects have been funded through this programme. This indicates that synthetic biology was not on the priority list set by the scientific committee from both institutes. Owing to lack of sufficient funding for research in general, research priorities are set to support a limited number of projects mainly from already established disciplines. A small number of synthetic biology-related research activities in France were funded through other French agencies, such as Genopole and the inter-EPST^16^ Bioinformatique (Remy et al., 2003; Yartseva et al., 2007).

With limitations in national funding, French scientists tend to get most of their support from pan-European programmes such as the European Research Council (ERC) and the European Commission (EC), for projects like PROBACTYS (NEST pathfinder), COBIOS and TARPOL.^17^ ANR has participated in various European-coordinated activities. It has been a partner in several ERA-NETs and a member of the European Science Foundation (ESF) although it did not support the ESF EUROSYNBIO call. ANR recognises that scientific progress on SB will not be welcomed by the public without addressing its effects on ELSI, although no SB ELSI project has yet been funded by ANR or CNRS. In the future, the interviewees believe that ELSI studies should be considered in the cooperation between these fields and other sciences (natural sciences, engineering, etc.). To deal with the interdisciplinary nature of synthetic biology, the French funding organisations reacted by setting up multidisciplinary teams. In ANR, one third of the applications were interdisciplinary and reviewed by multiple scientific committees.^18^

#### Switzerland

Switzerland is home to an active and productive research community in synthetic biology. Most projects have been funded by the Swiss National Science Foundation (SNSF). In 2007, SystemsX.ch was launched as the Swiss initiative in systems biology. It is a consortium composed of eight universities and three research institutions. The Swiss government provides €66.7 million (CHF 100 million) for four years for SystemsX.ch, which has to be matched by another €66.7 million (CHF 100 million) by the SystemsX.ch partner institutions. Funding is mainly for interdisciplinary, inter-institutional projects.^19^ Since issuing two calls for proposals, 14 research-, technology- and development projects have been funded, allocating about 80% of the funding between 2008 and 2011.^20^ Within the same measure, the Swiss government provides another €66.7 million (CHF 100 million) for the Department of Biosystems Science and Engineering (D-BSSE) in Basel, one of the most active SB research centres in Europe.^21^ It was founded to bring together research from multiple disciplines of natural sciences, engineering, computing and mathematics, with synthetic biology being one of three research foci. Three synthetic biology-related projects were funded by SystemsX.ch. The total budget reserved for SB is approx. €5.33 million (CHF 8 million) per year for a 4-year period from D-BSSE.

With a total annual budget of €480 million (CHF 719 million),^22^ SNSF is the leading provider of scientific research funding for basic research in all disciplines, including applied research. Many synthetic biology-related projects have been funded by SNSF since 2002.^23^ Most of these projects have been supported by the funding for investigator-driven bottom-up research. All projects funded by SystemsX.ch and SNSF address the scientific aspects of synthetic biology, but not its societal and ELSI aspects. The projects supported by SNSF are more directed toward basic research, whereas SystemsX.ch supports both fundamental and applied research.

Aiming to improve cooperation with other European countries, SNSF has been a partner with the EU framework research programmes since 1987 and has participated in several ESF calls, including the call of EUROSYNBIO.^24^

As an emerging field of research, synthetic biology is highly dependent upon innovations, many of which come from the interfaces of multiple scientific disciplines. Thus, expertise is required from a broad variety of disciplines. Although the interdisciplinary nature of synthetic biology might have posed problems for some funding organisations, this is not the case for SystemsX.ch. A typical research, technology and development project funded by SystemsX.ch is required to consist of researchers from at least two partner institutes.^25^ In SNSF, interdisciplinary projects will be evaluated by the Specialised Committee for Interdisciplinary Research when the proposed projects include research topics from two or more disciplines.^26^

#### Austria

Austria provided funding for several societal research projects despite the lack of national research and development (R&D) advances in synthetic biology. Among these research projects on social science, one project on biosafety issues of synthetic biology is funded by the Austrian Science Fund (FWF).^27^ Two others on the science–society interface and perception of synthetic biology are funded by the Austrian Research Promotion Agency’s (FFG)^28^ GEN-AU ELSI programme.^29^ In addition, the first European NEST project on safety and ethical aspects of synthetic biology was also initiated and coordinated by Austrian ELSI scientists.^30^

With a total annual funding of more than €100 million, FWF is the main funding body for basic science in Austria.^31^ It supports all branches of science and the humanities. Within its funding categories for scientific disciplines, the FWF’s applied principle of competition is based solely on the quality of the proposal. No SB related basic research proposal has been submitted to the FWF so far, showing the lack of interest of the Austrian R&D community in SB.

FFG is the national funding agency for industrial research and development in Austria.^32^ The scientific officer of FFG expressed the opinion that synthetic biology is at the beginning stage and that SB-related research is more about basic research, explaining the lack of activities at FFG. Among FFG’s science programmes, however, is the Austrian Genome Research Programme (GEN-AU), which funds basic research projects and ELSI research.^33^ However, owing to the lack of active synthetic biology research, as well as to the lack of funding resulting from the current economic situation, no special call has been issued for synthetic biology. So far, no natural science research proposal dealing with synthetic biology has been submitted to FFG. Interestingly, the funded SB-related projects are concerned solely with societal aspects (ELSI) (Cserer and Seiringer, 2009; Ganguli-Mitra et al., 2009; Kelle, 2009; Schmidt et al., 2009; Torgersen, 2009).

In Austria, R&D in synthetic biology is relatively sparse. Depending on how broadly synthetic biology is defined, we can identify a few research groups in Austria or none at all. Owing to the limited current SB research activities in Austria, the Austrian agencies consider funding at the national level to be sufficient for synthetic biology at present. Should Austrian scientists decide to start research in this field, funding resources do not seem to be a limiting factor.

As for future SB research proposals, the interdisciplinary nature of such projects is not seen as a problem for the Austrian funding agencies. The proposals submitted to FFG, for example, are required to be interdisciplinary in nature. As for FWF, the decision is made based solely on the quality of project applications, and the interdisciplinarity of a project is not seen as a hindrance.

#### The Netherlands

Several research funding organisations in the Netherlands may fund synthetic biology according to their strategic focus. The most relevant ones are probably NWO, STW and the Netherlands Genomics Initiative (Hooshangi and Bentley, 2008). The Netherlands Organisation for Scientific Research (NWO), with an annual budget of €550 million, has a programme on systems biology that is closely related to and could theoretically serve as a starting point for synthetic biology research funding.^34^ The Netherlands Genomics Initiative (Hooshangi and Bentley, 2008), established by the Dutch government in 2002, has a total budget of €280 million. It could also be a nucleus for future synthetic biology research funding.^35^ Additionally, three Dutch universities allocated a total of €60 million to centres for synthetic biology research. The Delft University of Technology, with its department of nanoscience, invested €35 million over ten years. The University of Groningen, with the new Centre for Synthetic Biology, invested €10 million over five years. Finally, the Eindhoven University of Technology, with its institute for complex molecular systems, allocated €15 million over ten years.

The major funding organisations more or less agree on their definition of synthetic biology as:

Synthetic biology is the engineering of biology: the synthesis of complex, biologically based (or inspired) systems which display functions that do not exist in nature. This engineering perspective may be applied at all levels of the hierarchy of biological structures – from individual molecules to whole cells, tissues and organisms. In essence, synthetic biology will enable the design of “biological systems” in a rational and systematic way. (EC, 2005)

One interviewee mentioned that he preferred a narrower definition of synthetic biology as “this will help to maintain the focus of the discipline within life science, leaving the ethical issues aside.”

Whereas NWO has not funded any projects in synthetic biology so far,^36^ a representative from STW mentioned that “two sub-programmes of the nanotechnology research programme^37^ of the Netherlands – NanoNed, the BioNanoSystems and the Chemistry and Physics of Individual Molecules – would provide a certain linkage.” However, no synthetic biology projects were funded within these programmes and no specific funding for synthetic biology has so far been available in the Netherlands. “One reason for this was that educating the public on synthetic biology needed to be done before specific funds can be set aside” explained one interviewee. “On the other hand,” argued one interviewee, “combining systems and synthetic biology would dilute the development in systems biology.”

As practical applications of synthetic biology are still out of reach, the interviewees agreed that “funding should be directed to fundamental research, although” one interviewee observed “a shift towards applied research can be seen at least in the nanotechnology-related subfield.”

Funding the investigation of societal aspects of emerging technologies has a long tradition in the Netherlands. NWO argued, however, that there are no specific funds within the life science section for ELSI analyses. Thus, the ELSI studies should be carried out via the societal section of NWO.^38^ Multidisciplinarity is an issue for funding agencies in the Netherlands, and it seems easier to address this at the Dutch Technology Foundation STW, which forces groups to form multidisciplinary consortia. In contrast, NWO is more structured along the traditional disciplines, making it more complicated to address these issues.

### Lack of private financial support in Europe

The European Commission has played an active role in fostering research in synthetic biology. In NEST a total of 18 SB research and policy projects were funded.^39^ The total funding for these projects was over €32 million, of which one fourth was contributed by the participating countries (Table 1).

Considering the public funding for overall research and development as a percentage of national GDP, some European countries invested the same or even more than the US. Data from OECD indicated that, in 2005, the overall expenditure on R&D by EU27 was €168 billion versus €257 billion by the US.^40^ Regarding publications in synthetic biology, up to November 2009, 31% of the publications (234 of 759) in the field were published by researchers in the European Union (EU27) versus 39% from US-based research groups (297 of 759) (Figure 1). The main difference between Europe and the US is the lack of commercial interest and investment in Europe. For example, the Bill and Melinda Gates Foundation recently donated €28 million ($42 million) to develop a synthetic form of artemisinin, an anti-malarial drug, in a collaboration between UC Berkeley and Amyris Biotechnologies.^41^Amyris was also awarded €47 million ($70 million) to develop biofuel by a consortium of venture capitalists.^42^ Craig Venter’s Synthetic Genomics Inc. (SGI) signed a multi-year research and development agreement with ExxonMobil Research and Engineering Company to develop next-generation biofuels using photosynthetic algae. Total funding for SGI in research and development activities and milestone payments could amount to more than €200 million ($300 million).^43^ Similar investments in Europe have not been disclosed, and probably do not exist. We can speculate about the reasons for these different investment activities. The nature of synthetic biology research seems to be similar in the USA and Europe, at least when discussed among scientists at international conferences. Nonetheless, US scientists apparently manage more easily to move their research into the realm of near-commercial activities. In the view of company representatives, US research in synthetic biology seems to be more application driven compared to European research. This was at least the argument for BP to invest €333 million ($500 million) in the Energy Bioscience Institute at UC Berkeley. According to a statement from BP, in the context of a European forum on synthetic biology organised by the European Commission in March 2010, they could not find an appropriate partner for their activities in Europe.

## 4. Conclusion

Our results show that the funding situation for synthetic biology varies considerably among European countries. Some countries have – in relation to their size – a considerable R&D community in SB, including the UK, France and Switzerland. In the Netherlands and Germany, the R&D community seems rather fragmented and less established than in the three aforementioned countries. Austria is the only country in our study that did not have an active R&D community under the label SB. The situation for SB ELSI research communities deviates from R&D in several ways. A few scattered ELSI groups working on SB are located in France, Switzerland and the Netherlands. Germany is home to a small, nascent community of ELSI researchers in SB. Additionally, Germany is the only country where some ELSI aspects of SB, in particular security issues for DNA synthesis, are driven by the private sector, namely the German DNA synthesis companies themselves. Austria, although lacking an R&D community, has several ELSI groups working on different aspects of SB, funded through European and national sources. The UK, in contrast, is the only country with an established funding scheme for R&D and ELSI (which, however, is sometimes impaired by the detached structure of the individual research councils). See Table 3 for details.

**Table 3.** Funding landscape for synthetic biology and its ELSI research in six European countries

	Synthetic biology (SB)		ELSI of SB		
Country	Community	Funding	Community	Funding	Link funding–community
Austria	not existing	potentially available	existing	available	SB: lack of Austrian SB community although funding would be available, ELSI: good
France	existing	hardly available	emerging	not available	general lack of funding
Germany	emerging	potentially available	existing	Available	community and funding hardly synchronised, more money available than spent
Netherlands	emerging	available	emerging	hardly available	SB: good, ELSI: funding difficulties
Switzerland	existing	available	emerging	not available	SB: good, ELSI: funding difficulties
UK	existing	available	existing	available	overall good situation, community and funding available and synchronised

The best funding situation for R&D and ELSI in SB can probably be found in the UK. France, although having an active R&D community, apparently provides insufficient funding for them, and even less for the ELSI community. Switzerland has a good funding situation for its active R&D community, but less so for its ELSI community. The Netherlands does provide some funding for its R&D community, but less so for its ELSI scientists in SB. While Austria has enough funding for synthetic biology research, no researchers have yet applied for these funds, and the only researchers receiving funding are working on societal aspects. In Germany, public funding agencies and the research community (both R&D and ELSI) appear to be sub-optimally linked together. While several R&D groups work on SB, the funding agencies commented that hardly any proposal is submitted to them through the bottom-up approach.

Most of the funding for synthetic biology in Europe is contributed by public sources. We can speculate that the increasing commercial prospects of synthetic biology will attract additional public funding for the technology-driven economy as well as funding from the private sector, which is still not the case in Europe.

The development of synthetic biology promises to provide a better understanding to answer basic questions about life. It also holds promise for useful applications, such as new energy, new biomaterials, and new medicines. All these involve different societal ramifications. This calls for conducting research on such societal ramifications and on ethical, legal and social issues (ELSI). Owing to the nature of synthetic biology, research on ELSI can hardly fit into any single discipline. Therefore, such work should also be conducted with an interdisciplinary perspective to build up new links between natural sciences and the social sciences and humanities (Rabinow and Bennett, 2009). With the exception of the UK, however, which is attempting to foster this interaction through its funding scheme (and a few cases on the provincial level in Germany), this interaction is mostly missing in Europe. The reasons for this lack of integration are country specific, reflecting for example a lack of an established ELSI community in SB (France, Switzerland, Netherlands), lack of funding opportunities (France), lack of an R&D community (Austria), or poor linkage of the available groups with established funding opportunities (Germany).

## Notes

1 See: EC, 2005 at www.synbiosafe.eu/uploads///pdf/EU-highlevel-syntheticbiology.pdf and EC, 2007 at http://cordis.europa.eu/nest/

2 See: www.gopubmed.org

3 See http://www.esf.org/activities/eurocores/programmes/eurosyntheticbiology/funding-agencies-and-contacts.html for list of countries that support the EUROCORES EUROSYNTHETIC BIOLOGY call.

4
http://www.bmbf.de/en/1056.php

5 See press release from 6 November 2009: http://idw-online.de/pages/de/news342927 and http://www.uni-marburg.de/aktuelles/news/2009a/0708b

6
http://www.bmbf.de/en/1237.php

7
http://www.bbsrc.ac.uk/publications/corporate/synthetic_biology.pdf

8
http://www.epsrc.ac.uk/AboutEPSRC/Plans/M3E.htm

9 See: “Synthetic Biology Sandpit: Collaboration Between EPSRC and US NSF” at http://www.epsrc.ac.uk/newsevents/news/2009/Pages/syntheticbiologysandpit.aspx

10 The UK Royal Society, together with the OECD and the US National Academies of Sciences co-organised a joint International Symposium on Opportunities and Challenges in the Emerging Field of Synthetic Biology in July 2009 in Washington, DC. See: http://sites.nationalacademies.org/PGA/stl/PGA_050738

11 In October 2009 the Wellcome Trust – the UK’s largest medical research charity – announced that it is offering teams of students stipends to enable them to enter the International Genetically Engineered Machine competition (iGEM) 2010. The stipend will provide promising undergraduates with hands-on experience in synthetic biology. See: http://www.wellcome.ac.uk/News/Media-office/Press-releases/2009/WTX056649.htm

12
http://www.sgm.ac.uk/news/consultations/sgmcon082.pdf

13 The review, by Andrew Balmer and Paul Martin, can be downloaded from http://www.syntheticbiologyethics.org

14
http://www.agence-nationale-recherche.fr/AAPProjetsOuverts?lngAAPId=198

15
http://www.cnrs.fr/en/aboutCNRS/key-figures.htm

16 EPST: Etablissements publics à caractère scientifique et technologique (French public institutions for science and technology).

17
http://www.agence-nationale-recherche.fr/documents/uploaded/2008/ANR-Annual-Report-2007.pdf

18
http://www.agence-nationale-recherche.fr/documents/uploaded/2008/ANR-Annual-Report-2007.pdf

19
http://www.systemsx.ch/about-us/what-is-systemsxch/

20
http://www.systemsx.ch/projects/systemsxch-projekte/rtd-projekte/?L=3

21 See: http://www.bsse.ethz.ch/

22
http://www.snf.ch/E/aboutus/facts/Pages/statistics.aspx

23
http://www.projectdb.snf.ch/WebForms/Frameset.aspx

24
http://www.snf.ch/E/international/Pages/default.aspx

25
http://www.systemsx.ch/about-us/what-is-systemsxch

26
http://www.snf.ch/E/current/Dossiers/Pages/interdisciplinary.aspx

27
http://www.idialog.eu/fwf

28
http://www.synbio.at

29
http://www.cisynbio.com

30
http://www.synbiosafe.eu

31
http://www.fwf.ac.at/en/index.asp

32
http://www.ffg.at/content.php?version=2

33
http://www.gen-au.at/artikel.jsp?id=781&type=news&lang=de

34 Health Council of the Netherlands, Advisory Council on Health Research, and Royal Netherlands Academy of Arts and Sciences. “Synthetic Biology: Creating Opportunities.” The Hague: Health Council of the Netherlands, 2008; publication no. 2008/19E. ISBN 978-90-5549-742-3.

35 See note 34, page 1.

36 However, support for Dutch individual projects within the ESF EUROSYNBIO call was ensured.

37 Among others, NanoNed aims at strengthening Dutch knowledge infrastructure through a comprehensive knowledge transfer approach, leading to industrial nanotechnology applications (processes, products, services, general industrial activities). Additionally, NanoNed devotes attention to societal output and the innovation process (source: http://www.nanoned.nl/valorisation.html).

38 Although not dealing with synthetic biology, the NGI-related Dutch Centre for Society and Genomics (CSG) analyses, assesses and improves the relationship between society and genomics research. The CSG might serve as a starting point for future projects dealing with ELSI of synthetic biology. See: http://www.society-genomics.nl

39
ftp://ftp.cordis.europa.eu/pub/nest/docs/5-nest-synthetic-080507.pdf

40
http://stats.oecd.org/Index.aspx?DatasetCode=CSP2009

41
http://berkeley.edu/news/media/releases/2004/12/13_gates.shtml

42
http://www.amyrisbiotech.com/index.php?option=com_content&task=view&id=50&Itemid=307

43
http://www.syntheticgenomics.com/media/press/71409.html

## References

[bibr1-0963662510393624] Bhattachary D. Calitz J.P. Hunter A. (2010) Synthetic Biology Dialogue. London: TNS-BMRB

[bibr2-0963662510393624] Cserer A. Seiringer A. (2009) “Pictures of Synthetic Biology: A Reflective Discussion of the Representation of Synthetic Biology (SB) in the German-language Media and by SB Experts,” Systems and Synthetic Biology 3(1–4): 27–35 19816797 10.1007/s11693-009-9038-3 PMC2759430

[bibr3-0963662510393624] European Commission (2005) “Synthetic Biology: Applying Engineering to Biology. Report of a NEST High-Level Expert Group.” EUR 21796 Luxembourg: Office for Official Publications of the European Communities

[bibr4-0963662510393624] European Commission (2007) “Synthetic Biology: A NEST Pathfinder Initiative.” EUR 22426 Luxembourg: Office for Official Publications of the European Communities

[bibr5-0963662510393624] Ganguli-Mitra A. Schmidt M. Torgersen H. Deplazes A. Biller-Andorno N. (2009) “Of Newtons and Heretics,” Nature Biotechnology 27(4): 321–2 10.1038/nbt0409-321 19352363

[bibr6-0963662510393624] Gaisser S. Reiss T. Lunkes A. Müller K.M. Bernauer H. (2009): “Making the most of synthetic biology. Strategies for synthetic biology development in Europe”. EMBO Rep 2009 August; 10(S1): S5–S8 19636305 10.1038/embor.2009.118 PMC2726001

[bibr7-0963662510393624] Hooshangi S. Bentley W.E. (2008) “From Unicellular Properties to Multicellular Behavior: Bacteria Quorum Sensing Circuitry and Applications,” Current Opinion in Biotechnology 19(6): 550–5 18977301 10.1016/j.copbio.2008.10.007

[bibr8-0963662510393624] Kelle A. (2009) “Ensuring the Security of Synthetic Biology: Towards a 5P Governance Strategy,” Systems and Synthetic Biology 3(1–4): 85–90 19816803 10.1007/s11693-009-9041-8 PMC2759433

[bibr9-0963662510393624] Mingardon F. Chanal A. López-Contreras A.M. Dray C. Bayer E.A. Fierobe H.-P. (2007) “Incorporation of Fungal Cellulases in Bacterial Minicellulosomes Yields Viable, Synergistically Acting Cellulolytic Complexes,” Applied and Environmental Microbiology 73(12): 3822–32 17468286 10.1128/AEM.00398-07 PMC1932714

[bibr10-0963662510393624] Rabinow P. Bennett G. (2009) “Synthetic Biology: Ethical Ramifications 2009,” Systems and Synthetic Biology 3(1–4): 99–108 19816805 10.1007/s11693-009-9042-7 PMC2759434

[bibr11-0963662510393624] Remy E. Mossé B. Chaouiya C. Thieffry D. (2003) “A Description of Dynamical Graphs Associated to Elementary Regulatory Circuits,” Bioinformatics 19(Suppl. 2): ii172–ii178 14534187 10.1093/bioinformatics/btg1075

[bibr12-0963662510393624] Schmidt M. Ganguli-Mitra A. Torgersen H. Kelle A. Deplazes A. Biller-Andorno N. (2009) “A Priority Paper for the Societal and Ethical Aspects of Synthetic Biology,” Systems and Synthetic Biology 3(1–4): 3–7 19816794 10.1007/s11693-009-9034-7 PMC2759426

[bibr13-0963662510393624] Torgersen H. (2009) “Synthetic Biology in Society: Learning from Past Experience?,” Systems and Synthetic Biology 3(1–4): 9–17 19816795 10.1007/s11693-009-9030-y PMC2759423

[bibr14-0963662510393624] Yartseva A. Klaudel H. Devillers R. Képès F. (2007) “Incremental and Unifying Modelling Formalism for Biological Interaction Networks,” BMC Bioinformatics 8: 433 17996051 10.1186/1471-2105-8-433 PMC2200675

